# Genome-Wide Identification and Expression Analysis of the Biotin Carboxyl Carrier Subunits of Heteromeric Acetyl-CoA Carboxylase in *Gossypium*

**DOI:** 10.3389/fpls.2017.00624

**Published:** 2017-05-01

**Authors:** Yupeng Cui, Yanpeng Zhao, Yumei Wang, Zhengjie Liu, Babar Ijaz, Yi Huang, Jinping Hua

**Affiliations:** ^1^Laboratory of Cotton Genetics, Genomics and Breeding, College of Agronomy and Biotechnology/Beijing Key Laboratory of Crop Genetic Improvement, China Agricultural UniversityBeijing, China; ^2^Research Institute of Cash Crop, Hubei Academy of Agricultural SciencesWuhan, China; ^3^Oil Crops Research Institute, Chinese Academy of Agricultural SciencesWuhan, China

**Keywords:** biotin carboxyl carrier subunit (*BCCP*), gene family, expression profile, *Gossypium*, phylogenetic analysis

## Abstract

Acetyl-CoA carboxylase is an important enzyme, which catalyzes acetyl-CoA’s carboxylation to produce malonyl-CoA and to serve as a committed step for *de novo* fatty acid biosynthesis in plastids. In this study, 24 putative cotton *BCCP* genes were identified based on the lately published genome data in *Gossypium*. Among them, 4, 4, 8, and 8 *BCCP* homologs were identified in *Gossypium raimondii, G. arboreum, G. hirsutum*, and *G. barbadense*, respectively. These genes were divided into two classes based on a phylogenetic analysis. In each class, these homologs were relatively conserved in gene structure and motifs. The chromosomal distribution pattern revealed that all the *BCCP* genes were distributed equally on corresponding chromosomes or scaffold in the four cotton species. Segmental duplication was a predominant duplication event in both of *G. hirsutum* and *G. barbadense.* The analysis of the expression profile showed that 8 *GhBCCP* genes expressed in all the tested tissues with changed expression levels, and *GhBCCP* genes belonging to class II were predominantly expressed in developing ovules. Meanwhile, the expression analysis for the 16 cotton *BCCP* genes from *G. raimondii, G. arboreum* and *G. hirsutum* showed that they were induced or suppressed by cold or salt stress, and their expression patterns varied among different tissues. These findings will help to determine the functional and evolutionary characteristics of the *BCCP* genes in *Gossypium* species.

## Introduction

Polyploidy genomes have undergone rapid changes in genome structures and expression of duplicated genes (Li et al., 2011). The tetraploid cotton species originated from an inter-specific hybridization event between progenitors of A-genome species, *G. arboreum* (A_2_), and D-genome species, *G. raimondii* (D_5_) (Cronn et al., 1999; Senchina et al., 2003; Wendel et al., 2009). In tetraploid cotton (2*n* = 52), both Upland cotton (*G. hirsutum*, AD_1_) and Sea island cotton (*G. barbadense*, AD_2_), chromosome numbers 1 to 13 are reserved for the A subgenome (At), while chromosome numbers 14 to 26 have reserved for the D subgenome (Dt) (Kohel, 1973). Upland cotton accounts for more than 90% output of production, and cultivated in more than 130 countries due to its higher yield and fiber quality. Moreover, cotton ranks sixth among the world oil crops, providing cooking oil extracted from its oil-rich seeds which could be used for food industry either.

In plants, oil is one of the major components of seeds, stored in cottonseed as triacylglycerol (TAG). The accumulation of seed oil is mainly provided by TAG biosynthesis (Li et al., 2011; Troncosoponce et al., 2011; Hu et al., 2013; Sood and Chauhan, 2015). The pathway and related genes of lipid biosynthesis have been studied in many plants (Beisson et al., 2003; Bates et al., 2009; Yin et al., 2013; Jang et al., 2015). In *Arabidopsis*, at least 24 enzymes or subunits are involved in the plastid fatty acid synthetic pathway, which are encoded by 46 nuclear genes and one plastid gene (Beisson et al., 2003). Among them, acetyl-CoA carboxylase (ACCase) (E.C. 6.4.1.2) is an important enzyme, which catalyze acetyl-CoA to produce malonyl-CoA, and can be served as a committed step for *de novo* fatty acid biosynthesis in plastid (Sasaki and Nagano, 2004; Fukuda et al., 2013; Ran et al., 2015; Sood and Chauhan, 2015). In dicotyledon and non-graminaceous monocotyledon plants, plastidial ACCase is a multi-subunit complex comprised of four different polypeptides (biotin carboxyl carrier protein, BCCP; biotin carboxylase, BC; α- and β-carboxyltransferase subunits, α-CT and β-CT) with the exception of rapeseed plastid, which contains multifunctional ACCase comprised of a large multifunctional polypeptide (Elborough et al., 1996; Schulte et al., 1997; Sasaki and Nagano, 2004; Cui et al., 2017).

Among the subunits of heteromeric ACCase, BCCP subunit interacts with BC and accepts a bicarbonate ion when ATP is converted to ADP, thus it can be served as the donor of activated carboxyl group, then the complex (BCCP-biotin) transfers the ion to CT, and finally produces malonyl-CoA in fatty acid biosynthesis (Ke et al., 2000; Sasaki and Nagano, 2004; Chen et al., 2012; Jang et al., 2015). Previous studies revealed that BCCP subunits have variable protein lengths, and low similarity (Qiao and Liu, 2007), while each of BCCP subunits contains a biotinylated domain located at the C-terminal region, harboring a highly conserved motif (CIIEAMKLMNEIE) (Qiao and Liu, 2007; Xuan et al., 2015). Studies reported that BCCP subunit was redundant, and encoded by small gene family (Elborough et al., 1996; Reverdatto et al., 1999; Thelen et al., 2001). In rapeseed (*Brassica napus*), there were at least six copies of *BCCP* homolog, which can be divided into two distinct classes based on the comparison of amino acids (AAs) and nucleotide sequences (Elborough et al., 1996). In *Arabidopsis, BCCP* is encoded by two paralogous genes, *AtBCCP1* and *AtBCCP2* (Thelen et al., 2000, 2001), both share approximately 30% AA identity. *AtBCCP1* was constitutively expressed in all the tissues, while *AtBCCP2* was predominantly expressed in developing seeds (Thelen et al., 2000).

A *BCCP* homolog in Upland cotton, *GhBCCP1*, was constitutively expressed in all the tested tissues, indicating its indispensable role in cotton growth and metabolism. In addition, southern blotting result showed that there were several copies of *BCCP* genes in the cotton genome (Qiao and Liu, 2007), and overexpression of *GhBCCP1* in cotton could increase cottonseed oil content (Cui et al., 2017). The recent availability of the completed genome sequences of *G. raimondii* (Paterson et al., 2012; Wang et al., 2012), *G. arboreum* (Li et al., 2014), *G. hirsutum* (Li et al., 2015; Zhang T. et al., 2015), and *G. barbadense* (Liu X. et al., 2015; Yuan et al., 2015) provide us new opportunity to identify and characterize *BCCP* genes in cotton.

In the present study, we performed a comprehensive analysis of *BCCP* genes in the four cotton species with the phylogenetic relationship, gene structure, conserved motifs, chromosomal distribution and expression profiling. Moreover, we assessed the paralogous and orthologous relationships of the four cotton species. The identification and comprehensive study of *BCCP* genes will provide valuable information for further study of the biological function and evolution in cotton.

## Materials and Methods

### Data Search and Analysis for *BCCP* Family Members in *Gossypium*

The genome databases of *G. arboreum* (A2, BGI_V1.0), *G. raimondii* (D5, JGI_v2.1), *G. hirsutum* acc. TM-1 (NBI_V1.1), and *G. barbadense* acc.3-79 (NBI_V1.0) were downloaded from the CottonGen website^1^ (Yu et al., 2014).

The published AA sequences of BCCP for *Arabidopsis*, rapeseed, and soybean (*Glycine max*) were obtained from the NCBI (Supplementary Table 1). In order to identify all the candidate *BCCP* genes of four *Gossypium* species, several local BLAST searches (BlastP and tBlastN programs with default parameters) were performed using the BCCP protein sequences of *Arabidopsis*, rapeseed, and soybean as queries. Subsequently, the Pfam^2^ (Finn et al., 2014) and SMART^3^ (Letunic et al., 2015) databases were used to confirm each candidate of the *BCCP* gene family. Finally, in order to further verify the reliability of the initial results, all candidates were analyzed to confirm the presence of the conserved biotinyl domain using the InterProScan program^4^ (Quevillon et al., 2005). The online ExPASy tool^5^ was used to predict the theoretical MW (molecular weight) and *pI* (isoelectric point) of the BCCP proteins. Subcellular localization was predicted using the WoLF PSORT^6^ (Horton et al., 2007) and TargetP 1.1^7^ (Emanuelsson et al., 2007), and the online Chlorop 1.1 Server^8^ was used to predict the presence of chloroplast transit peptides (cTP) in protein sequences. The 5′ upstream region, a 2-kb genomic DNA sequence of each gene was extracted from the genome database, and then subjected to the plantCARE database^9^ for a *cis*-element scan.

### Phylogenetic Analysis, Gene Structure Prediction and Conserved Motif Identification

Multiple sequence alignment was performed using Clustal X version 2.0 program (Larkin et al., 2007) with default parameters. Subsequently, MEGA version 5.0 software (Tamura et al., 2011) were employed to construct an unrooted phylogenetic tree, using the method of Neighbor Joining with pairwise deletion option, poisson correction model and uniform rates (rates among sites). Bootstrap tests with 1000 replicates were carried out to evaluate the statistical reliability of phylogenetic tree. The gene structure of *BCCP* genes were obtained through comparing the genomic sequences and their predicted coding sequences using the Gene Structure Display Server (GSDS) tool^10^ (Hu et al., 2015). BCCP protein sequences in *G. arboreum, G. raimondii, G. hirsutum*, and *G. barbadense* were submitted to online MEME program^11^ (Bailey et al., 2009) for identification of conserved protein motifs. The MEME parameters were as follows: any number of repetitions, maximum number of motifs: 4, and optimum motif widths from 6 to 80 AA residues.

### Analysis of Chromosomal Location and Gene Duplication

The chromosomal localization of each *BCCP* gene in *G. arboreum, G. raimondii, G. hirsutum*, and *G. barbadense* was deduced based on the available genomic information at the CottonGen database^12^ (Yu et al., 2014). Mapchart version 2.2 software (Voorrips, 2002) was used to visualize the distribution of *BCCP* genes on the chromosomes. Gene duplication events were defined when the following conditions were fulfilled: (1) the length of aligned sequence covered more than 80% of the longer gene, (2) the identity of the aligned regions was bigger than 80%, and (3) only one duplication event was taken into counted for tightly linked genes (Jiang et al., 2013; Wei et al., 2013; Dong et al., 2016). According to the chromosomal locations of *BCCP* genes, two types of gene duplications (tandem duplication and segmental duplication) were recognized.

### Estimating *K*_a_/*K*_s_ Ratio for Duplicated Gene Pairs

The *BCCP* duplicated gene pairs of *G. arboreum, G. raimondii, G. hirsutum*, and *G. barbadense* were firstly aligned by Clustal X version 2.0 program (Larkin et al., 2007). Subsequently, synonymous substitution (*K*_s_) and non-synonymous substitution (*K*_a_) were calculated using the DnaSP version 5.0 software (DNA polymorphism analysis) (Rozas et al., 2003). Finally, the selection pressure for each gene pair was assessed by the *K*_a_/*K*_s_ ratio.

### RNA-Seq Data Analysis

For the expression analysis, the public expression data for various tissues (root, stem, and leaf), floral tissue (petal) and ovule tissues at different developmental stages (5, 10, 20, 25, and 35 DPA) in *G. hirsutum* TM-1 were obtained from Zhang T. et al. (2015), according to the identified *GhBCCP* ID. The expression data were gene-wise normalized and the heatmap for gene expression patterns was illustrated with the software MultiExperiment Viewer (MeV).

### Plant Materials and Stress Treatments

Cotton seedlings of *G. arboreum* L. Var. Shixiya 1, *G. raimondii*, and *G. hirsutum* L. acc TM-1 were grown in a temperature-controlled chamber with a photoperiod of 16 h light and 8 h darkness at 28°C. Three seedlings at trefoil stage were exposed to low temperature (4.0°C) for 24 h and salt (150 mM NaCl) for 24 h, respectively. Then the roots, stems, and leaves were sampled and frozen in liquid nitrogen immediately, and stored at -80°C for RNA isolation. Three biological repeats were performed for each treatment.

### RNA Isolation and Quantitative Reverse Transcriptase-Polymerase Chain Reaction (qRT-PCR)

Approximately 1 μg RNA was used for first-strand cDNAs synthesis with the PrimeScript 1st Strand cDNA Synthesis Kit (TakaRa, Dalian, China) following the manufacturer’s instructions. Gene-specific primer pairs were designed using Primer version 5.0 software based on CDSs of the *BCCP* genes. The sequences of the primer pairs are listed in Supplementary Table 2. The qRT-PCR analysis was performed with the SYBR Premix Ex Taq (TakaRa, Dalian, China) following the manufacturer’s instructions. In all the qRT-PCR analyses, the cotton *UBQ7* gene was used as an internal reference. Each sample was run with three biological replicates and three technical replicates on an ABI 7500 real-time PCR System (Applied Biosystems, Foster City, CA, USA). The thermal cycle applied was as follows: 95°C for 30 s, followed by 40 cycles of denaturation at 95°C for 5 s and annealing and elongation at 60°C for 35 s. The relative expression levels (RQ) were calculated according to the 2^-ΔΔCt^ method (Livak and Schmittgen, 2001).

## Results

### Genome-Wide Identification of *BCCP* Genes in *Gossypium*

The genome-wide identification of *BCCP* genes have been performed on the basis of four cotton genome sequences, *G. raimondii* (Paterson et al., 2012), *G. arboreum* (Li et al., 2014), *G. hirsutum* (Zhang T. et al., 2015), and *G. barbadense* (Liu X. et al., 2015). BLASTP and BLASTN programs were used to search the candidate *BCCP* genes from the four cotton species genome databases with the query sequences of *Arabidopsis* (2), rapeseed (6), and soybean (2) BCCP proteins. Among the 6 *BCCP* genes in rapeseed (Elborough et al., 1996), only 2 (*BnpBP4* and *BnpBP6*) coded completed proteins and were used. Subsequently, Interproscan (Quevillon et al., 2005) and SMART were used to verify the biotinyl domain (CIIEAMKLMNEIE) of the retrieved sequences. The results showed that a total of 24 *BCCP* genes, which contained biotinyl domain, were identified in the four cotton species genomes (**Table 1**). Among them, 4 were predicted in *G. raimondii*, 4 in *G. arboreum*, 8 in *G. hirsutum*, and 8 in *G. barbadense*. The predicted *BCCP* genes, *GrBCCP1*-*GrBCCP4, GaBCCP1*-*GaBCCP4, GhBCCP2*-*GaBCCP8*, and *GbBCCP1*-*GaBCCP8* were numbered based on their chromosomal location. Though the size of *G. arboreum* genome was about twofold than the *G. raimondii* (Paterson et al., 2012; Wang et al., 2012; Li et al., 2014), each of the two diploid species has 4 *BCCP* genes. The length of 8 BCCP proteins from the two diploid cotton species varied from 244 to 295 AAs, and their predicted molecular weights and *pI* values were within the ranges of 25.50–31.57 kDa and 5.03–8.74, respectively. For *G. hirsutum*, the GhBCCP proteins were varied from 282 AA of GhBCCP1 to 313 AA of GhBCCP3, their molecular weights ranged between 29.43 kDa of GhBCCP5 to 33.41 kDa of GhBCCP2, and their *pI* values were distributed in a range from 4.91 of GhBCCP5 to 8.66 of GhBCCP3. For *G. barbadense*, the length of 8 GbBCCP proteins varied from 57 AA of GbBCCP4 to 515 AA of GbBCCP6, the MW ranged from 6.22 kDa of GbBCCP4 to 54.42 kDa of GbBCCP6, and the *pI* values were between 4.65 of GbBCCP4 and 8.90 of GbBCCP8. Compared with the length of BCCP homologs reported in other plants (Thelen et al., 2000; Gu et al., 2011), GbBCCP4, GbBCCP6, and GbBCCP8 were less than 200 AA or more than 350 AA.

**Table 1:** The information of *BCCP* genes in four *Gossypium* species^a^.

Gene name	Gene identifier	Genomics position	CDS	Exons	Protein			Subcellular location		Predicted cTP length^c^
					Size (AA)	Mw (kDa)	pI	WolF PSPORT	TargetP	
*GrBCCP1*	Gorai.006G011100.1	Chr06:2420726–2423347	855	7	284	30.36	8.59	Chlo: 14	C 0.946/1	81
*GrBCCP2*	Gorai.010G135200.1	Chr10:30546370–30557469	858	6	285	29.82	6.62	Chlo: 13	C 0.929/1	62
*GrBCCP3*	Gorai.012G049400.1	Chr12:6445631–6448034	885	6	294	31.2	5.71	Chlo: 14	C 0.989/1	79
*GrBCCP4*	Gorai.013G132300.1	Chr13:34624954–34632153	852	7	283	29.55	4.91	Chlo: 13	C 0.825/2	61
*GaBCCP1*	Cotton_A_38676	CA_chr8:1977503–1980631	735	5	244	25.5	5.03	Chlo: 9, cyto: 2, nucl_plas: 2	M 0.530/4	-
*GaBCCP2*	Cotton_A_14712	CA_chr11:102621972–102624096	888	7	295	31.57	8.74	Chlo: 14	C 0.926/1	81
*GaBCCP3*	Cotton_A_18292	CA_chr12:118242679–118244704	888	6	295	31.26	5.99	Chlo: 14	C 0.987/1	86
*GaBCCP4*	Cotton_A_23281	CA_chr13:53936790–53943440	855	6	284	29.57	5.35	Chlo: 13	C 0.906/1	59
*GhBCCP1^b^*	Gh_D06G1228 /EF555556.1	D06:32123415–32132928	849	7	282	29.45	6.62	Chlo: 13	C 0.921/1	62
*GhBCCP2*	Gh_A05G3209	A05:83883408–83886052	936	7	311	33.41	6.13	Chlo: 13	C 0.987/1	79
*GhBCCP3*	Gh_A06G1022	A06:51531079–51547092	942	5	313	33.3	8.66	Chlo: 13	C 0.940/1	62
*GhBCCP4*	Gh_A09G0096	A09:2421313–2423450	855	7	284	30.34	8.64	Chlo: 14	C 0.966/1	81
*GhBCCP5*	Gh_A13G0950	A13:50745148–50751938	852	7	283	29.43	4.91	Chlo: 14	C 0.895/1	34
*GhBCCP6*	Gh_D04G0397	D04:6284714–6286725	885	6	294	31.19	5.71	Chlo: 14	C 0.987/1	79
*GhBCCP7*	Gh_D09G0093	D09:2459463–2461601	855	7	284	30.34	8.59	Chlo: 14	C 0.946/1	81
*GhBCCP8*	Gh_D13G1202	D13:35856022–35862746	852	7	283	29.54	5.03	Chlo: 13	C 0.887/1	63
*GbBCCP1*	Gbscaffold265.5.0	At05:84945093–84948190	873	7	290	30.79	5.99	Chlo: 14	C 0.988/1	79
*GbBCCP2*	Gbscaffold3613.1.0	At06:49989074–49989989	636	4	212	22.15	6.6	Chlo: 10, nucl_plas: 2, cyto: 1	M 0.530/4	-
*GbBCCP3*	Gbscaffold13314.13.0	At09:2811384–2813784	846	7	281	30.05	8.64	Chlo: 14	C 0.966/1	78
*GbBCCP4*	Gbscaffold2855.10.0	At12:11698494–11701028	174	4	57	6.22	4.65	Chlo: 5, cyto: 5, extr: 2, nucl: 1	-	-
*GbBCCP5*	Gbscaffold1797.14.0	At13:54571562–54579207	732	5	243	25.07	4.85	Chlo: 10, extr: 2, mito: 1	C 0.277/5	63
*GbBCCP6*	Gbscaffold9097.20.0	Dt04:7081253–7086755	1548	12	515	54.42	5.19	Chlo: 14	C 0.987/1	79
*GbBCCP7*	Gbscaffold258.1.0	Dt13:36288436–36291900	714	6	237	24.56	4.47	-	-	-
*GbBCCP8*	Gbscaffold2694.3.0	Scaffold2694:201086–206379	1536	9	511	53.35	8.9	Chlo: 6, cyto: 4, nucl: 3	M 0.536/4	62

*^a^Gr, Ga, Gh, and Gb represented the genome data of *G. raimondii, G. arboreum, G. hirsutum* TM-1, and *G. barbadense*, respectively.*

*^b^Already exist in NCBI.*

*^c^There has not a chloroplast transit peptide.*

*WoLF PSORT predictions: chlo, chloroplast; cyto, cytosol; nucl, nucleus; plas, plasma membrane.*

*TargetP predictions: C, chloroplast; M, mitochondrion; - (any other location); values indicate score (0.00–1.00) and reliability class (1–5), and best class is 1.*

Multiple sequence alignments of 24 BCCP proteins from the four cotton species showed that the C-terminal region was conserved, and a typical biotinyl domain was existed (Supplementary Figure 1). In addition, the result of multiple sequence alignments showed that GbBCCP4 only contains C-terminal sequence (Supplementary Figure 1). Due to the small protein length of GbBCCP4, we were unable to analyze it in the subsequent research.

Protein subcellular localization is important for understanding the function of genes (Chou and Shen, 2007). According to the Wolf PSORT assessment, the result of the signal peptide prediction showed that the N-terminal of 23 BCCP proteins of the four cotton species carried cTPs (**Table 1**). Meanwhile, the subcellular localization of 23 BCCP proteins was predicted again by TargetP software, the result showed that GaBCCP1, GbBCCP2, and GbBCCP8 proteins were located in the mitochondria, GbBCCP7 protein could not predict its subcellular location since it was not start with methionine (Supplementary Figure 1), the rest of 19 proteins were located in the chloroplast. Based on the predicted subcellular location results, the cTPs length of 20 BCCP proteins was predicted using the ChloroP 1.1. It was showed that their length ranged from 34 to 86 AA (**Table 1**), implying that those BCCP proteins are chloroplast-located proteins. The cTPs may facilitate the BCCP precursor entering from cytosol to chloroplast (Gu et al., 2011).

### Phylogenetic, Gene Structure and Motif Analysis of BCCP Proteins in Cotton

**FIGURE 1:**
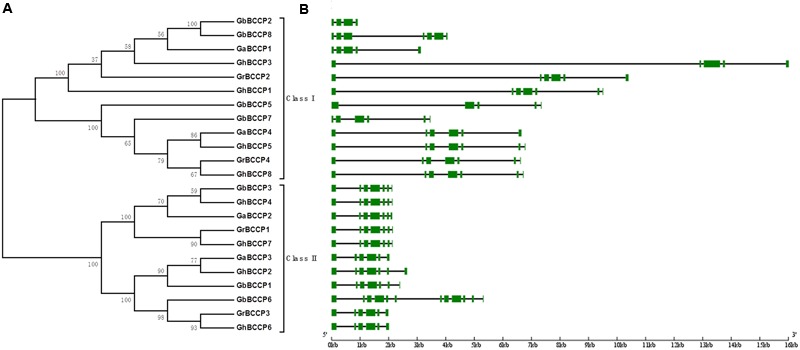
**Phylogenetic tree and gene structure of the *BCCP* gene family in *Gossypium*. (A)** The phylogenetic tree of all BCCP proteins in four *Gossypium* species was constructed using Neighbor-Joining method and the numbers at nodes represent bootstrap support values from 1000 replicates. **(B)** The exon/intron structure of *BCCP* genes in four *Gossypium* species. The green boxes represent exons and the black lines represent introns.

To assess the evolution of the BCCP homologs in *G. arboreum, G. raimondii, G. hirsutum*, and *G. barbadense*, 23 predicted full-length BCCP proteins were aligned to construct phylogenetic tree using a neighbor-joining (NJ) method (**Figure 1A**). Meanwhile, the phylogenetic trees were reconstructed with minimal evolution (ME) and maximum likelihood (ML) methods (Supplementary Figure 2). The trees produced by the two methods above showed less difference with the tree produced by NJ method, suggesting those three methods were largely consistent with each other, and the NJ tree was suitable for further analysis. According to the NJ tree, 23 BCCP proteins from the four cotton species were divided into two classes designated classes I and II (**Figure 1A**). This classification was consistent with *BCCP* genes in other plant species (Thelen et al., 2000; Li et al., 2010). Class I contained 12 members, composed of 2 members from *G. raimondii*, 2 from *G. arboreum*, 4 from *G. hirsutum*, and 4 from *G. barbadense*. Class II contained 11 members, 2, 2, 4, and 3 members in *G. raimondii, G. arboreum, G. hirsutum*, and *G. barbadense*, respectively.

Gene structure analysis is a significant method to study genetic evolution. We calculated the number of exons and introns in *BCCP* family members by comparing the genomic sequences with the predicted coding sequences in the four cotton species, and created *BCCP* structure in cotton. As shown in **Figure 1B**, gene length in class I was longer than that in class II. The code length of gene members in class I ranged from 951 to 16,013 bp, while in class II ranged from 2,000 to 3,000 bp, except for *GbBCCP6*, which contained 5,502 bp. The exons/intron distribution patterns in class I genes were various. The exons numbers ranged from four of *GbBCCP2* to nine of *GbBCCP9.* However, the exons/intron distribution patterns of class II genes were conserved except for *GbBCCP6* which had 12 exons. For example, six exons were found in *GrBCCP3, GaBCCP3*, and *GhBCCP6*, and seven exons in each of *GrBCCP1, GaBCCP2, GhBCCP2, GhBCCP4, GhBCCP7, GbBCCP1*, and *GbBCCP3*. Four conserved motifs were identified from the 23 cotton BCCP proteins using the MEME motif research tool (Supplementary Figure 3), and Supplementary Table 3 listed the length and sequence information of these four motifs. Motif 1 was the biotinyl motif, and it was present in all *BCCP* genes. Although motifs 2–4 did not belong to any known functional domains based on the searches using interproscan database, motifs 2 and 3 were primarily present in the C-terminal regions. Moreover, motif 4 was only present in GrBCCP3, GaBCCP3, GhBCCP2, GhBCCP6, GbBCCP1, and GbBCCP6 proteins (Supplementary Figure 3A). The presence of the same type of conserved motifs might indicate similar function among cotton *BCCP* genes.

### Orthologous Relationships among the Four Cotton Species

**FIGURE 2:**
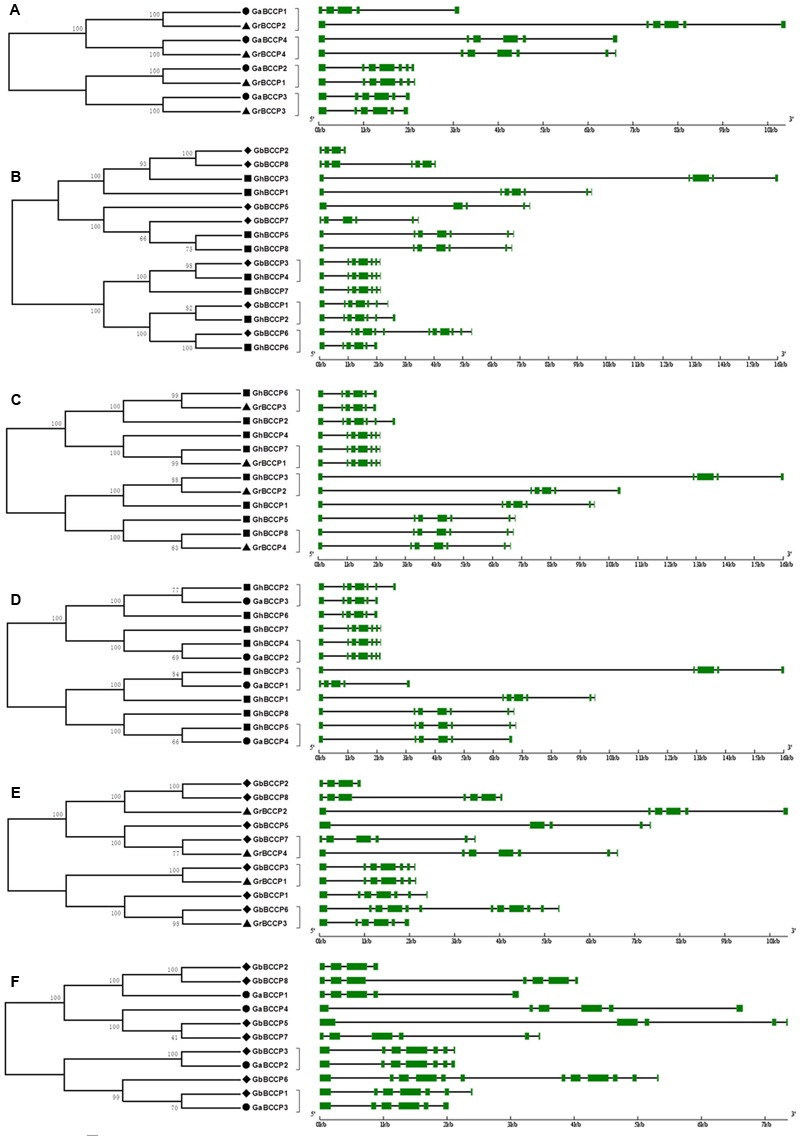
**Phylogenetic relationships and gene structure of *BCCP* genes in and between *G*. *raimondii, G*. *arboreum, G*. *hirsutum*, and *G. barbadense*. (A)** The phylogenetic tree and gene structure of *BCCP* genes in *G. raimondii* and *G. arboreum.* **(B)** The phylogenetic tree and gene structure of *BCCP* genes in *G*. *hirsutum* and *G. barbadense*. **(C)** The phylogenetic tree and gene structure of *BCCP* genes in *G*. *hirsutum* and *G. raimondii*. **(D)** The phylogenetic tree and gene structure of *BCCP* genes in *G*. *hirsutum* and *G. arboreum*. **(E)** The phylogenetic tree and gene structure of *BCCP* genes in *G. barbadense* and *G. raimondii*. **(F)** The phylogenetic tree and gene structure of *BCCP* genes in *G. barbadense* and *G. arboreum*. The *BCCP* genes from *G*. *raimondii, G*. *arboreum, G*. *hirsutum* and *G. barbadense* were marked with black triangles, black dots, black squares, and black rhombuses, respectively. Exons were represented by green boxes and introns by black lines.

In order to reveal the orthologous relationships of BCCP genes between the four cotton species, the protein sequences of 4 *GrBCCP* genes, 4 *GaBCCP* genes, 8 *GhBCCP* genes, and 7 *GbBCCP* genes were applied to construct six unrooted phylogenetic trees (**Figure 2**). The results showed that there were 20 pairs of orthologous genes among the four cotton species, since they were in the terminal branches with high bootstrap values. Among them, four orthologous gene pairs in the two diploid cotton (*G. raimondii* and *G. arboreum*) (**Figure 2A**), and there were three pairs of orthologous genes in the two allotetraploid cotton (*G. hirsutum* and *G. barbadense*) (**Figure 2B**). Four pairs of orthologous genes in *G. hirsutum* and *G. raimondii* were identified (**Figure 2C**), and four pairs in *G. hirsutum* and *G. arboreum* were found (**Figure 2D**). While, there were three pairs of orthologous genes from *G. barbadense* and *G. raimondii* (**Figure 2E**), two pairs in *G. barbadense* and *G. arboretum* (**Figure 2F**). The orthologous relationships among the four cotton species were displayed in Supplementary Figure 4. As expected, gene structures of orthologous pairs were almost identical with only minor differences with the exception of *GbBCCP6*/*GhBCCP6* and *GbBCCP6*/*GrBCCP3* (**Figures 2B,E**). However, based on the others were divergent apparently, the orthologous relationships of them could not be confirmed. In addition, a total of eleven pairs of paralogous genes in the four cotton species were found (Supplementary Figure 5), since the *BCCP* genes from the same genome were in the terminal branches of the phylogenetic trees. Among them, two pairs of paralogous genes in respective genome of *G. raimondii* and *G. arboreum*, and there were four paralogous pairs in *G. hirsutum* and three in *G. barbadense*.

### Chromosomal Location and Gene Duplication

**FIGURE 3:**
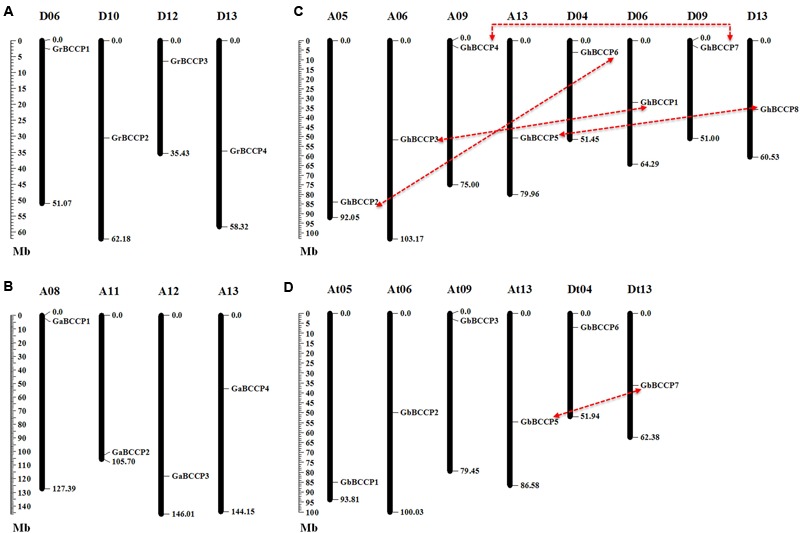
**Localization of *BCCP* genes in the four cotton species.** Twenty-two *BCCP* genes were mapped on different chromosomes in *G*. *raimondii* **(A)**, *G*. *arboreum* **(B)**, *G*. *hirsutum* **(C)**, and *G. barbadense* **(D)**. Only the chromosomes where *BCCP* genes were mapped are shown. Possible duplicated genes were connected by lines. The scale represents the megabases (Mb).

Based on the coordinate of each *BCCP* gene on the chromosomes, the chromosomal distribution images of *BCCP* genes in *G. raimondii, G. arboreum, G. hirsutum*, and *G. barbadense* were generated. In the four cotton species, the *BCCP* genes were distributed uniformly, one gene on each chromosome or scaffold (**Figure 3**). In *G. raimondii*, one *BCCP* gene was found in each of chromosome 6, 10, 12, and 13 (**Figure 3A**). In *G. arboreum*, only one gene was in each of chromosome 8, 11, 12, and 13 (**Figure 3B**). There were 8 *GhBCCP* genes in *G. hirsutum*, 4 genes were assigned to A subgenome and 4 to D subgenome, respectively (**Figure 3C**). In A subgenome of *G. hirsutum*, each of chromosome 5, 6, 9, and 13 had only one gene, and only one in each of chromosome 4, 6, 9, and 13 in D subgenome of *G. hirsutum*. The 7 *GbBCCP* genes were mapped on six *G. barbadense* chromosomes and one scaffold (**Figure 3D**). Each only single *BCCP* gene was localized on chromosome 5, 6, 9, and 13 in At subgenome, and one gene on each of chromosomes 4 and 13 in Dt subgenome. *GbBCCP8* was ambiguous and could not be assigned to either subgenome, but it merely anchored on unmapped scaffold.

Genomic changes, including chromosomal rearrangement, gene duplication and expression change of genes, often occurred during the formation of polyploidy species (Cronn et al., 1999). And gene duplication events were considered to play an important role in the amplification of gene families (Cannon et al., 2004; Maere et al., 2005). We investigated the gene duplication events of *BCCP* genes in the four cotton species, respectively. Firstly, we used the following criteria (Zhou et al., 2004): the alignment length covered >70% of the longer aligned gene, and the AA identity between the sequences was >70%, to identify gene duplication events. A total of nine segmental duplication events were found (Supplementary Table 4). Among them, two duplication gene pairs were, respectively, found in genome of *G. raimondii* and *G. arboretum*, and four in *G. hirsutum*. In *G. barbadense*, only one gene pairs were found. All those duplicated gene pairs were located on different chromosomes, suggesting all of them were segmental duplication events. Subsequently, we employed a stringent criteria, the alignment length covered >80% of the longer gene, and the identity of the aligned regions >80%, (Jiang et al., 2013; Wei et al., 2013; Dong et al., 2016). Under this rule, only 4 and 1 segmental duplication events were found in *G. hirsutum* and *G. barbadense*, respectively (**Table 2**), comparable to the number generated with 70% cover length and AA identity criterion. These results suggested that segmental duplication played crucial roles in the expansion of the *BCCP* gene family in the two allotetraploid cotton species.

**Table 2:** *K*_a_/*K*_s_ analysis of the duplicated gene pairs in *GhBCCPs* and *GbBCCPs*.

Species	Duplicated gene 1	Duplicated gene 2	*K*_a_	*K*_s_	*K*_a_/*K*_s_	Purifying selection	Duplicate type
*G. hirsutum*	*GhBCCP1*	*GhBCCP3*	0.033	0.038	0.868	Yes	Segmental
	*GhBCCP2*	*GhBCCP6*	0.029	0.054	0.537	Yes	Segmental
	*GhBCCP4*	GhBCCP7	0.014	0.04	0.35	Yes	Segmental
	*GhBCCP5*	GhBCCP8	0.014	0.061	0.23	Yes	Segmental
*G. barbadense*	*GbBCCP5*	GbBCCP7	0.06	0.06	1	Yes	Segmental

During the progress of evolution, the duplicated gene pairs might have undergone three alternative fates, i.e., non-functionalization, neofunctionalization, and subfunctionalization (Lynch and Conery, 2000). To explore different selective constrains on duplicated *BCCP* genes in *G. hirsutum* and *G. barbadense*, the *K*_a_/*K*_s_ ratio for each pair of duplicates were calculated (**Table 2**). Generally, *K*_a_/*K*_s_ > 1 indicates positive selection (accelerated evolution), *K*_a_/*K*_s_ = 1 indicates neutral selection (the genes are pseudogenes), while *K*_a_/*K*_s_ < 1 indicates negative or purifying selection (the functional constraint of the genes) (Liu et al., 2014; Dong et al., 2016). In this study, the *K*_a_/*K*_s_ ratios for four duplicated *BCCP* gene pairs were less than 1, suggesting that the *BCCP* genes from *G. hirsutum* have mainly experienced purifying selection pressure. While in the case of *G. barbadense*, the one *BCCP* duplicated gene pair (*GbBCCP5/GbBCCP7*) with a ratio = 1, indicating neutral selection. Those results reflected that the function of the duplicated *BCCP* genes in the two cotton species did not diverge much during subsequent evolution, and the maintenance of function in *G. hirsutum BCCP* genes might contributed to purifying selection.

### Cotton *BCCP* Family Relationships with Other Plants *BCCPs*

**FIGURE 4:**
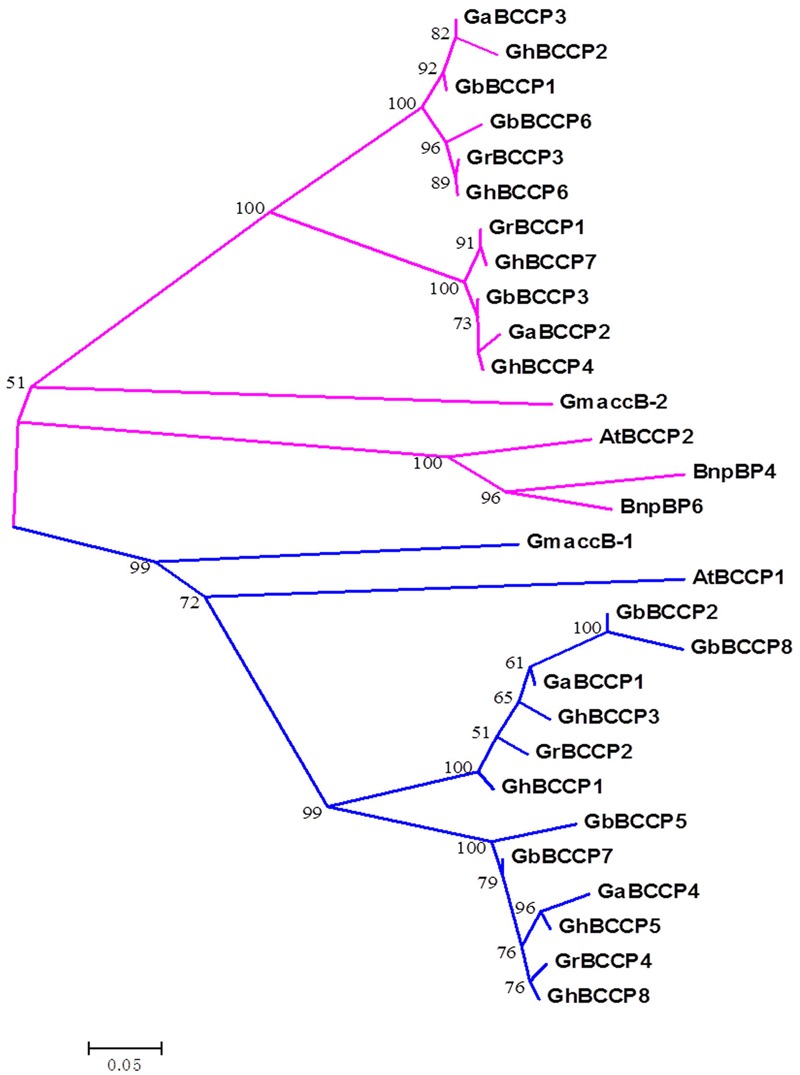
**Phylogenetic analysis of *BCCP* genes in *Gossypium* and other plants.** The unrooted phylogenetic tree containing 4 *G. raimondii* (*GrBCCP*), 4 *G*. *arboreum* (*GaBCCP*), 8 *G*. *hirsutum* (*GhBCCP*), 7 *G. barbadense* (*GbBCCP*), 2 *Arabidopsis* (*AtBCCP*), 2 soybean (*GmaccB*), and 2 rapeseed (*BnpBP*). *BCCP* genes were constructed using the neighbor-joining method with 1000 replicates.

Phylogenetic tree was served as a common method to reveal homologous relationships and evolutionary root of BCCPs from species. To detect the evolutionary relationships of *BCCP* genes, a NJ phylogenetic tree was constructed with the alignments of BCCP protein sequences of four cotton species, *Arabidopsis*, rapeseed and soybean BCCP proteins (**Figure 4**). Compared with the other three species, BCCP proteins from the four cotton species had higher relative coefficient, suggesting a closer relationship. It also appeared that cotton *BCCPs* fell into two evolutionary classes defined by the two *Arabidopsis* BCCPs, which was consistent with *BCCP* genes in *Brassicaceae* oilseeds (Thelen et al., 2000). We surmised that they might have distinct roles in fatty acid biosynthesis. The phylogenetic tree clearly indicated that the *BCCP* genes (*GrBCCP2* and *GrBCCP4* of *G. raimondii*; *GaBCCP1* and *GaBCCP4* of *G. arboreum; GhBCCP1, GhBCCP3, GhBCCP5*, and *GhBCCP8* of *G. hirsutum; GbBCCP2, GbBCCP5, GbBCCP7*, and *GbBCCP8* of *G. barbadense*) were closely related to the *AtBCCP1* and *accB-1* of soybean (**Figure 4**), implying that they may have similar function(s).

### Expression Patterns of *GhBCCP* Genes in *G. hirsutum* TM-1

**FIGURE 5:**
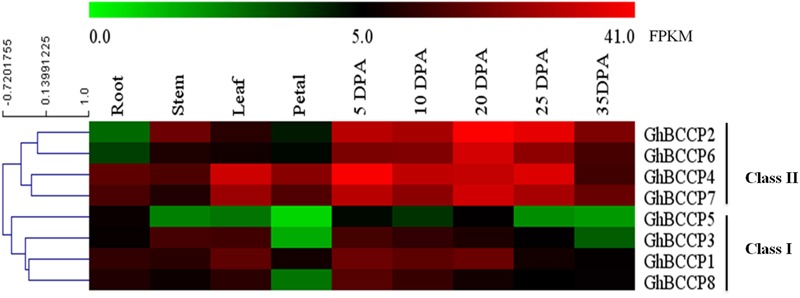
**Expression analysis of *ChBCCP* genes in *G. hirsutum* acc TM-1 across 9 tissues.** The RNA-Seq expression profiles of *G. hirsutum* acc. TM-1 (Zhang T. et al., 2015) were used to identify the expression levels of *ChBCCP* genes. FPKM represents fragments per kilobase of exon model per million mapped reads. DPA, days post anthesis.

In order to understand the role of *GhBCCP* genes better in *G. hirsutum* acc. TM-1, temporal expression patterns of these genes were analyzed using the public expression data, which contained vegetative tissues (root, stem, and leaf), floral tissue (petal), and ovule tissues at different developmental stages (5, 10, 20, 25, and 35 DPA) (Zhang T. et al., 2015). As shown in **Figure 5**, the expression of *GhBCCP* genes could be detected in all of the investigated tissues of TM-1, indicating that the *GhBCCP* genes were involved in multiple progresses during the development of the cotton. The heat map also revealed that the *GhBCCP* genes showed specific spatial expression patterns. Based on the hierarchical clustering analysis, the eight *GhBCCP* genes could be clustered into two classes (classes I and II) (**Figure 5**). Compared with the *GhBCCP* genes in class I, class II genes exhibited higher transcript abundance in the ovules at the five developmental stages. In addition, the expression levels of genes in class II were up-expressed in the ovules compared with that in vegetative and floral tissues (**Figure 5**). During the ovule developmental stages, *GhBCCP* genes in class II had higher expression in ovules at early and middle developmental stages (5–25 DPA), but relatively lower expressions in later developmental stages (35 DPA), indicating that they might contribute to fatty acids accumulation mainly occurring before the maturation stage in upland cotton seeds. Furthermore, the expression patterns of four duplicated gene pairs (**Table 2**) were slightly different in present research. Two duplicated gene pairs such as *GhBCCP2*/*GhBCCP6* and *GhBCCP4*/*GhBCCP7* were clustered together, and shared highly similar expression patterns in all the tested tissues (**Figure 5**). The expression of *GhBCCP1*/*GhBCCP3* was similar in all investigated tissues except for in petals and 35 DPA ovules. However, the duplicated gene pairs of *GhBCCP5*/*GhBCCP8* were divergent, which might be caused by the significant variation in gene regulation after the duplication events.

### Expression Patterns of *BCCP* Genes under Abiotic Stresses

Salt and cold stresses are the two serous environmental stresses that most plants might encounter during their growth and developmental processes. Precious study reported that *GhBCCP1* gene has been induced by cold stress for 24 h (Cui et al., 2017). However, little is known about the function of cotton *BCCP* genes responsing to salt and cold stresses. Gene promoter is the control center of gene transcription, and the *cis*-elements in gene promoter regions could provide some evidence for dissection of gene functions in stress response (Zhou et al., 2013). In present study, we identified all the *cis*-regulatory elements in the promoter regions of 16 cotton *BCCP* genes from *G. raimondii, G. arboreum*, and *G. hirsutum* (Supplementary Table 5), and there were eight putative environmental stress-related elements in the promoter regions of the three cotton *BCCP* genes (Supplementary Table 6). Although there were no special items of salt responsive element and only one low temperature responsive *cis*-element existed in the PLACE database, some *cis*-elements might respond to multiple environment stimuli (Higo et al., 1999). The results showed that each *BCCP* gene in the three cotton species contained more than three environmental stress-related elements (Supplementary Table 6), indicating that these *BCCP* genes might the signal transduction of the cotton response to salt and cold stresses.

**FIGURE 6:**
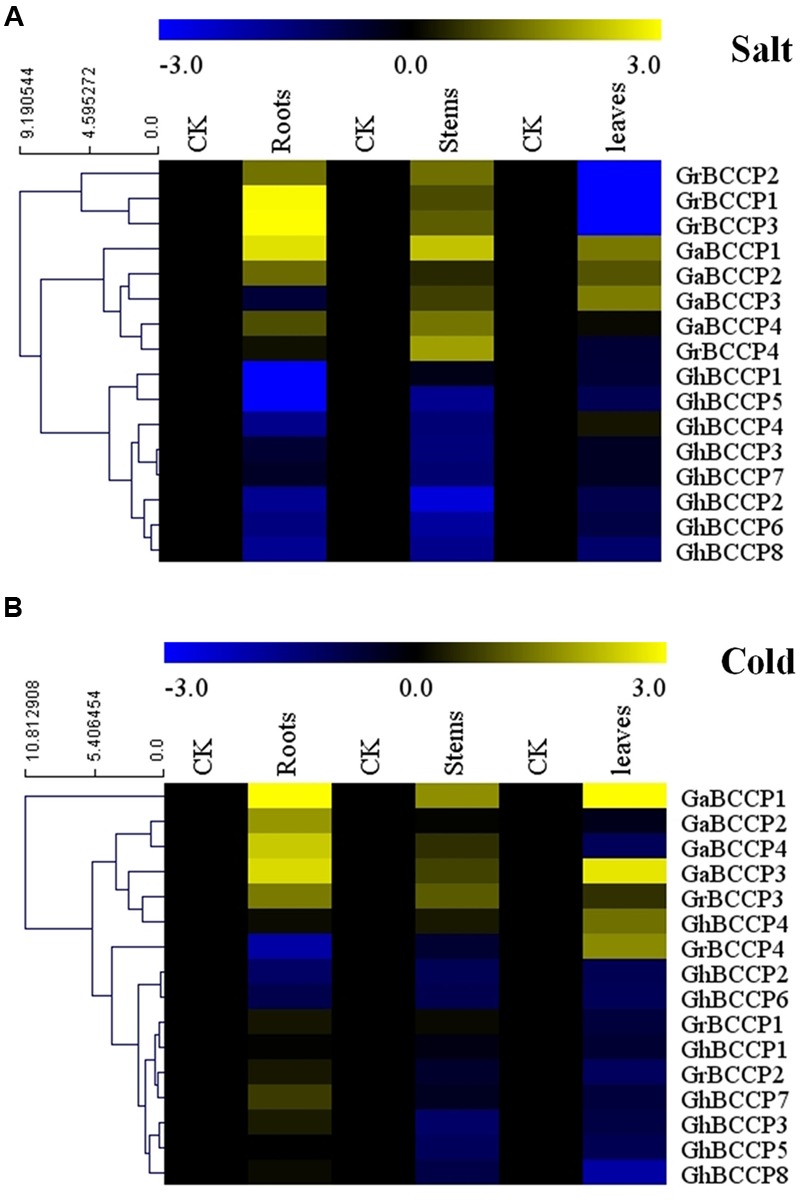
**Expression patterns of 16 *BCCP* genes in three representative tissues of *G. raimondii, G. arboreum*, and *G. hirsutum* response to salt stress and cold stress. (A)** Expression levels 16 cotton *BCCP* genes under salt stress, **(B)** Expression levels 16 cotton *BCCP* genes under cold stress. The color bar represents the relative signal intensity values.

To investigate the expression patterns of *BCCP* genes in different tissues under salt and cold (4°C) stresses, roots, stems, and leaves in each of *G. raimondii, G. arboreum* var Shixiya 1, and *G. hirsutum* acc TM-1 at trefoil stage were treated and used for RNA extraction. The expression levels of *BCCP* genes responsive to salt and cold stresses were shown in **Figure 6**, and it showed that these *BCCP* genes from the three cotton species expressed diversely under both stresses. For the salt stress (**Figure 6A**), in roots, six *BCCP* genes in cotton showed up-regulated expression after salt treatment for 24 h, *GrBCCP4* showed insignificantly up-regulated expression, while the rest genes expressed down-regulation. In stems, all the genes in *G. raimondii* and *G. arboreum* were up-regulated expression under salt stress, and seven of eight *GhBCCP* genes showed down-regulated. However, only a few up-regulated *BCCP* genes were found in leaves compared with roots and stems. Three *GaBCCP* genes showed down-regulated expression in leaves, *GaBCCP4* and *GhBCCP4* showed no significantly change compared with that control, others were down-regulated. For the cold stress (**Figure 6B**), the heat map showed that *GrBCCP3, GaBCCP1, GaBCCP2, GaBCCP3, GaBCCP4*, and *GhBCCP7* were induced up-regulation in root. And only four genes were suppressed in roots compared with stem and leaves. In stem, only *GrBCCP3, GaBCCP1, GaBCCP3*, and *GaBCCP4* were found up-regulated after cold stress for 24 h. In leaves, five genes were induced and the others were suppressed by cold stress for 24 h (**Figure 6B**). Notably, there were four *BCCP* genes (*GrBCCP3, GaBCCP1, GaBCCP2*, and *GaBCCP4*) up-regulated expression in roots under both salt and cold stresses. And also found four *BCCP* genes (*GrBCCP3, GaBCCP1, GaBCCP3*, and *GaBCCP4*) expressed up-regulated in stems under both salt and cold stresses. In leaves, two cotton *BCCP* genes (*GaBCCP1* and *GaBCCP3*) were induced by both salt and cold treatment.

## Discussion

The BCCP subunit is one of the four subunits of ACCase that catalyzes the irreversible carboxylation of acetyle-CoA to produce malonyl-CoA in fatty acid biosynthesis (Thelen et al., 2000; Fukuda et al., 2013; Jang et al., 2015). Many studies have indicated that modifying the *BCCP* genes could change the oil content of transgenic *Arabidopsis* (Thelen and Ohlrogge, 2002; Li et al., 2011). Cotton is a significant oilseed crop, and cottonseeds are important source of edible oil and potential industrial raw material (Cui et al., 2017). However, only *GhBCCP1* gene has already been genetically manipulated for oil improvement in cottonseed (Cui et al., 2017). In present study, a comprehensive set of 24 *BCCP* genes was identified from the available genomes of the four cotton species. Undoubtedly, these identified cotton *BCCP* genes will provide candidate genes for the gene engineering of fatty acid biosynthesis in plants.

The results of present study revealed the details of 24 *BCCP* genes in the four cotton species. Among them, 4 were predicted in *G. raimondii*, 4 in *G. arboreum*, 8 in *G. hirsutum*, and 8 in *G. barbadense*, which indicated that *BCCP* genes in each of the two tetraploid cotton genomes were the sum of the two diploid cotton genomes. The predicted full-length 23 BCCP proteins were categorized into two classes, 12 genes in class I, and 11 in class II (**Figure 1**), and this classification was also presented in **Figure 4**. As shown in **Figure 4**, all the cotton *BCCP* genes could be divide into the same two classes according to the two *AtBCCP* genes in *Arabidopsis* (**Figure 4**), this was consistent with previous report in *Brassicaceae* oilseeds (Thelen et al., 2000). Each *GrBCCP* gene or *GaBCCP* gene in each of the diploid cotton species corresponded to two *GhBCCP* genes in the tetraploid cotton belonging to one homologous *BCCP* group, this was consistent with whole genome duplication events occurred during the evolution of *Gossypium* (Li et al., 2014). According to the distribution of intron/exon in *BCCP* genes, the gene in the same class shared the similar introns/exons structure and exon numbers (**Figure 1B**), but the gene length in class I were longer than class II, and the number of introns/exons in the terminal branch of phylogenetic tree were still different in some of the pairs. These findings indicated some introns loss, or introns gain, might have occurred during the *BCCP* structure evolution in the four cotton species. The prediction of motifs showed that all the BCCP proteins contained the biotinly domain (CIIEAMKLMNEIE) at C-terminal (Supplementary Figure 3A), but GbBCCP2 was one exception, which only harbored CIIEAMKLMNEIE sequence at C-terminal (Supplementary Figure 1) and could not presented motif 1 in Supplementary Figure 3A. Functional domains analysis indicated that the biotinyl domain of ACCasee is to transfer CO_2_ from one subsite to another allowing carboxylation reaction (Jitrapakdee and Wallace, 2003; Gu et al., 2011).

Gene duplication plays an important role in the process of plant genomic and organismal evolution, and gene duplication events contain tandem duplication, segmental duplication, transposition events and whole-genome duplication (Flagel and Wendel, 2009). In present study, we investigated gene duplicated events in order to further understand the expansion mechanism of *BCCP* genes in the four cotton species. Four duplicated gene pairs were identified in *G. hirsutum*, and one pair was found in *G. barbadense*. Among them, three segmental duplicated gene pairs, *GhBCCP1*/*GhBCCP3, GhBCCP5*/*GhBCCP8, GbBCCP5*/*GbBCCP7*, belonged to the class I, and the remaining two segmental duplicated gene pairs, *GhBCCP2*/*GhBCCP6* and *GhBCCP4*/*GhBCCP7*, belonged to the class II. These results showed that the expansion of *GhBCCP* genes and *GbBCCP* genes in class I were mainly caused by the segmental duplication. Duplicated genes might have undergone three different fates, the result showed the *K*_a_/*K*_s_ ratios for four duplicated *GhBCCP* gene pairs were less than 1, suggesting that these genes from *G. hirsutum* have mainly experienced purifying selection pressure. Gene expression patterns could provide useful clues for understanding these genes function. Based on these genes expression patterns in different tissues of TM-1 or response to salt and cold stresses performed in the study, the four *GhBCCP* duplicated gene pairs varied significantly. It was inferred that the functions of the four duplicated gene were different after duplication, and their fates could be described as neofunctionalization. These findings also further supported the assertion that expression divergence of duplicated genes is often the first step in the functional divergence, and this can increase the chance of duplicated genes being retained in a genome (Zhang, 2003).

Salt and cold stresses are the serious environmental stresses affecting the growth and yield of plants in many places of the world. Salt stress may increase the reactive oxygen species and damage the integrity of cell membrance (Zhu, 2002), and cold stress mainly alter the lipidic fluidity of membranes (Kargiotidou et al., 2008). Previous studies revealed that fatty acid synthesis related genes were induced or repressed by salt stress (Im et al., 2002; Zhang et al., 2009; Zeng et al., 2016) or cold stress (Liu et al., 2014; Liu W. et al., 2015; Zeng et al., 2016; Cui et al., 2017). In our study, the expression patterns of the 16 *BCCP* genes in the three cotton species (**Figure 6**) revealed that these genes were widely involved in responding to salt and cold stresses. Three of four *GrBCCP* genes (*GrBCCP1, GrBCCP2*, and *GrBCCP3*) were significantly up-regulated in response to salt stress in roots, suggesting these genes may be required to maintain certain activity of ACCase in cotton plants under salt treatment. Conversely, total of the four *GrBCCP* genes (*GrBCCP1, GrBCCP2, GrBCCP3*, and *GrBCCP4*) were down-regulated in leaves after salt treatment for 24 h (**Figure 6A**). The different expression patterns of *GrBCCP* genes responded to salt stress in roots and leaves might be associated with the fact that both tissues by themselves were distinct in structure and functions (Qing et al., 2009; Campo et al., 2014). Some cotton *BCCP* genes showed the same expression patterns in the same tissue after salt or cold stress, being either induced or suppressed, suggesting that these cotton *BCCP* genes were co-expressed in response to salt and cold stresses. However, a few of cotton *BCCP* genes in the same tissue responded to salt stress presented different expression patterns compared to cold stress. For instance, *GaBCCP3* was up-regulated in root by cold stress, while it was down-regulated by salt stress. This indicated that two sets of cotton *BCCP* genes were separately involved in cold and salt stresses. All these results implied that the signaling network responded to abiotic stress in plants was complicated (Zhang L. et al., 2015).

In short, the *BCCP* gene family in *G. raimondii, G. arboreum, G*. *hirsutum*, and *G. barbadense* were identified and comprehensive analyzed using bioinformatics methods, and all of these results provided valuable clues in future efforts to identify specific gene functions for *BCCP* gene family and gene physiological roles among *Gossypium* species.

## Author Contributions

Conceived and designed the experiments: JH. Performed the experiments and analyzed the data: YC and YZ. Maintained the experimental platform and performed bench work: YW. Attended discussion and part of experiments: ZL, BI, and YH. Contributed reagents/materials/analysis tools: JH. Prepared the manuscript: YC. Edited and revised the manuscript: JH.

## Conflict of Interest Statement

The authors declare that the research was conducted in the absence of any commercial or financial relationships that could be construed as a potential conflict of interest.

## Footnotes

1 https://www.cottongen.org/

2 http://pfam.xfam.org/

3 http://smart.embl-heidelberg.de/

4 http://www.ebi.ac.uk/Tools/pfa/iprscan/

5 http://web.expasy.org/compute_pi/

6 http://www.genscript.com/wolf-psort.html

7 http://www.cbs.dtu.dk/services/TargetP/

8 http://www.cbs.dtu.dk/services/ChloroP/

9 http://bioinformatics.psb.ugent.be/webtools/plantcare/html/

10 http://gsds.cbi.pku.edu.cn/

11 http://meme-suite.org/tools/meme

12 https://www.cottongen.org/data/download/genome
