# Supplementary material for: Genome-Wide Identification and Expression Analysis of the Biotin Carboxyl Carrier Subunits of Heteromeric Acetyl-CoA Carboxylase in Gossypium
Source: Front Plant Sci. 2017 May 1;8:624. doi: 10.3389/fpls.2017.00624 (PMC5410604; doi:10.3389/fpls.2017.00624)
Supplement: Supplementary file 1 [file Data_Sheet_1.docx]

**Supplementary Material:**

**Genome-wide identification and expression analysis of the biotin carboxyl carrier subunits of heteromeric acetyl-CoA carboxylase in *Gossypium***

Yupeng Cui ^1^, Yanpeng Zhao ^1^, Yumei Wang^2^, Zhengjie Liu ^1 ¶^, Babar Ijaz ^1^, Yi Huang^3^, and Jinping Hua ^1*^

*Corresponding authors, E-mail: jinping_hua@cau.edu.cn

Other authors’ email addresses are as follows: Y. C. [(yupeng851026@163.com);](mailto:(yupeng851026@163.com);) Y. Z. (yanpeng_zhao@cau.edu.cn); Y. W. ([yumeiwang001@126.com](mailto:yumeiwang001@126.com)); Z. L. (lzj1022@163.com); B. I. (babar_ijaz@cau.edu.cn); Y. H. (huangyi@oilcrops.cn)

**Supplementary Figures and Tables**

**
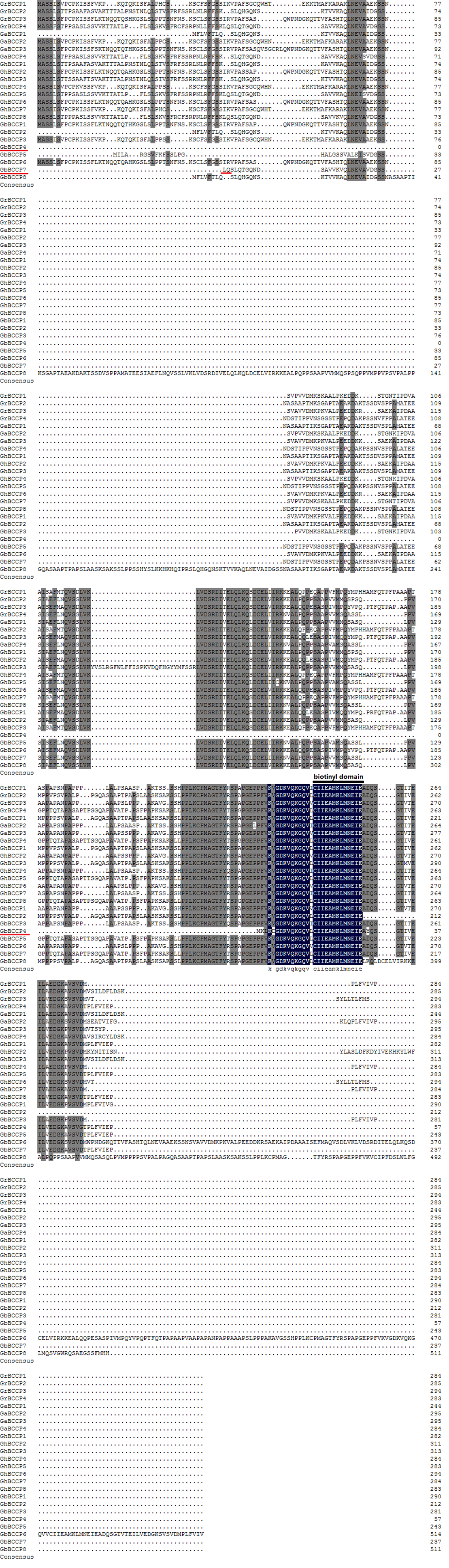
**

**Supplementary Figure 1. Multiple alignment analysis of 24 BCCP proteins from *G. raimondii,* *G. arboreum*, *G*. *hirsutum* and *G. barbadense*.**

**
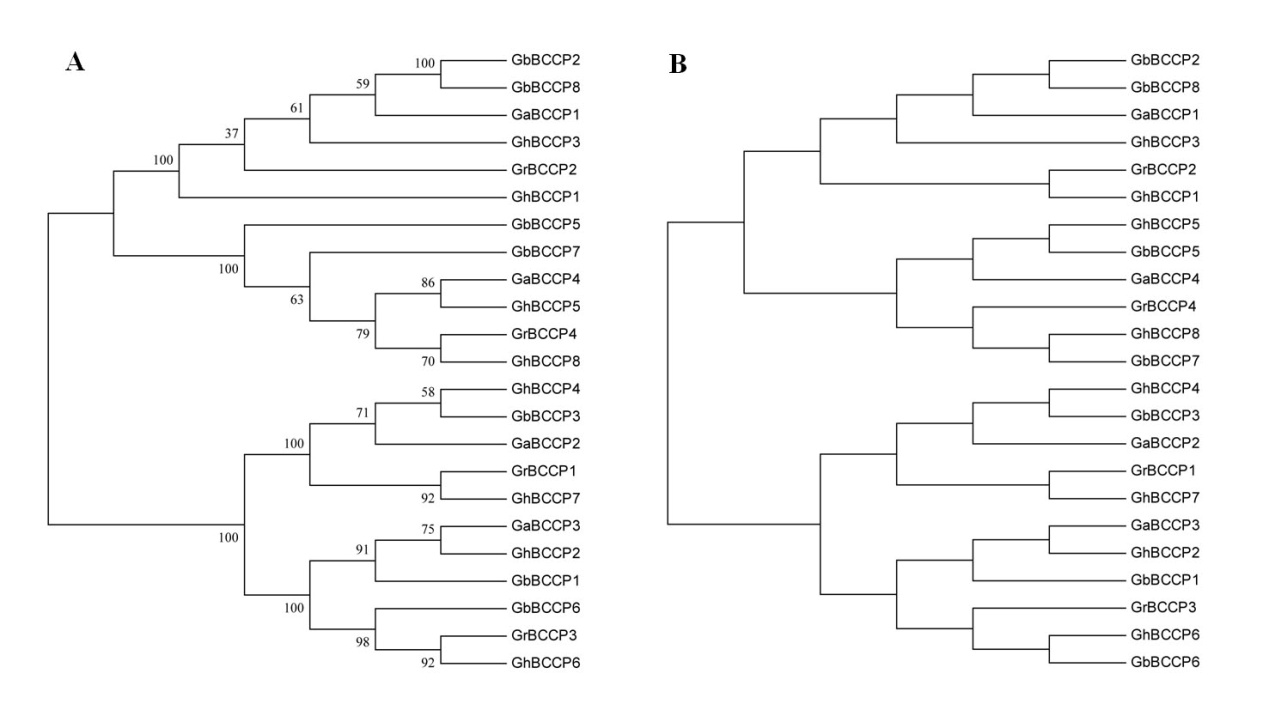
**

**Supplementary Figure 2. The phylogenetic tree of 23 BCCP proteins in four *Gossypium* species.** The unrooted phylogentic tree was constructed using Minimum Evolution method (A) and Maximum likelihood method (B). The numbers at nodes represent bootstrap support values from 1000 replicates.

**
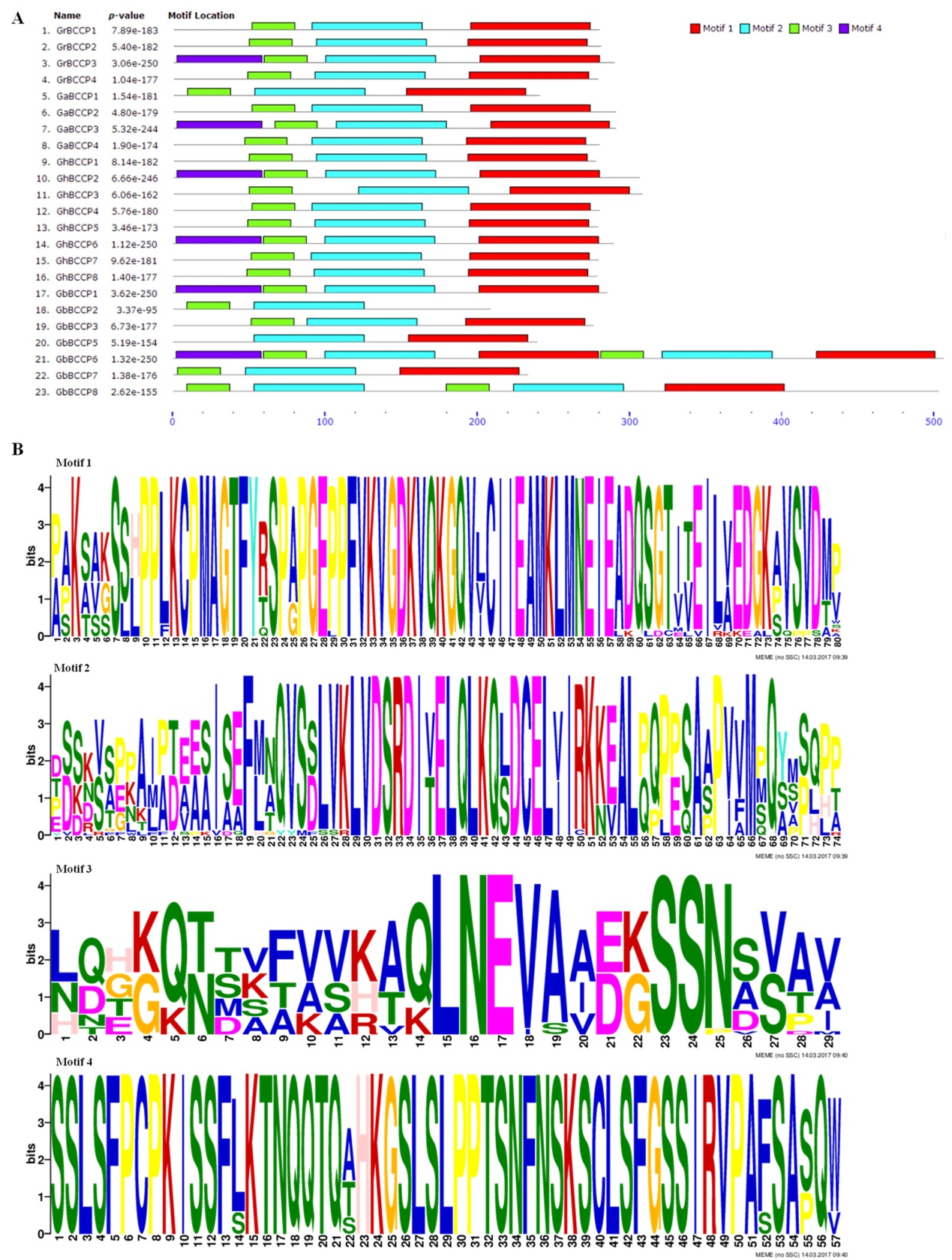
**

**Supplementary Figure 3. The conserved motifs (A) and sequence logos (B) of cotton BCCP proteins in the four cotton species were identified using the MEME search tool.** Each motif was indicated with a specific color.

**
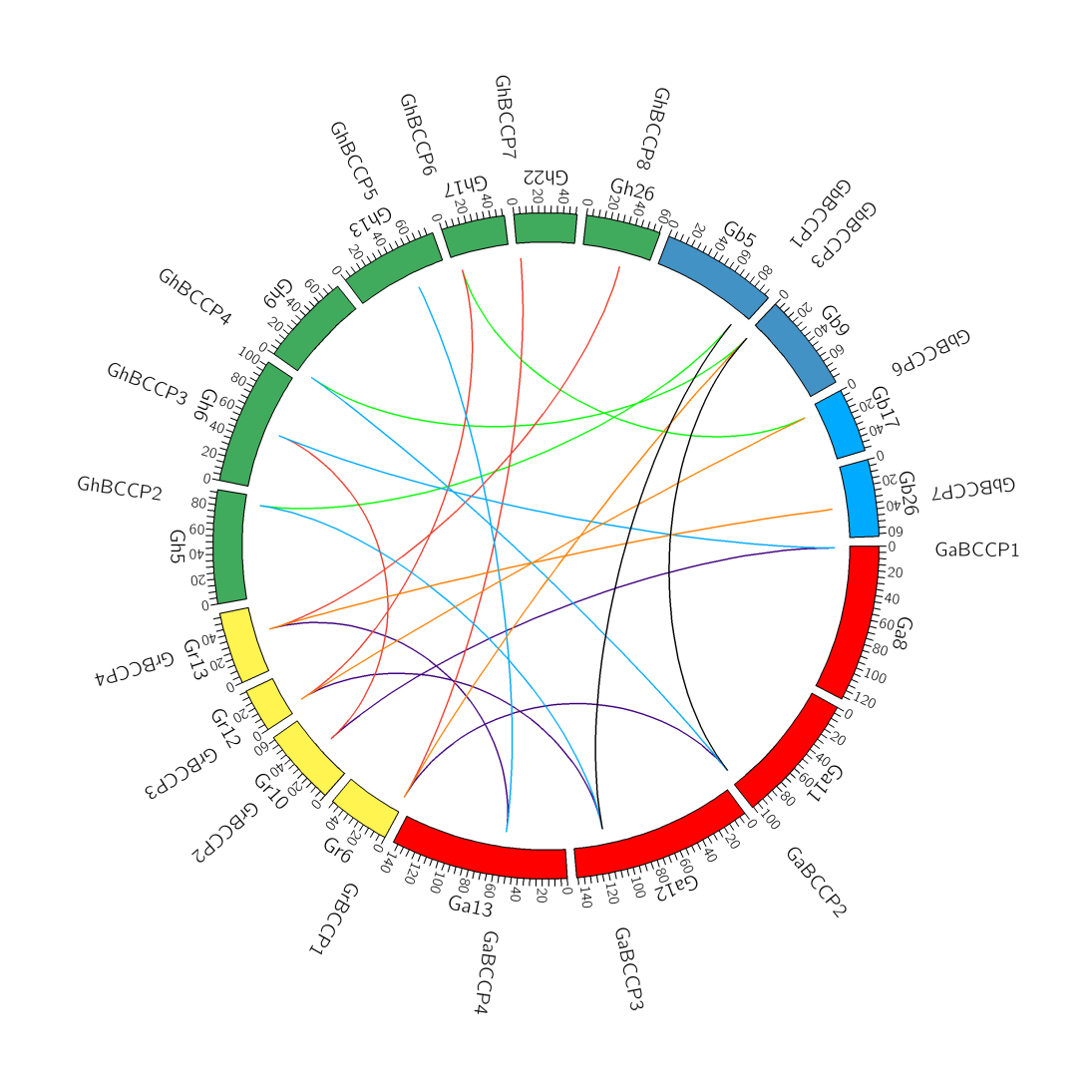
**

**Supplementary Figure 4. Locations and orthologs of *BCCP* genes in *G. raimondii,* *G. arboreum*, *G*. *hirsutum* and *G. barbadense*.** The picture was made by Circos software. The chromosomes of *G. raimondii,* *G. arboreum*, *G*. *hirsutum* and *G. barbadense* were shown with different colors and labeled as Gr, Ga, Gh, Gb respectively.

**
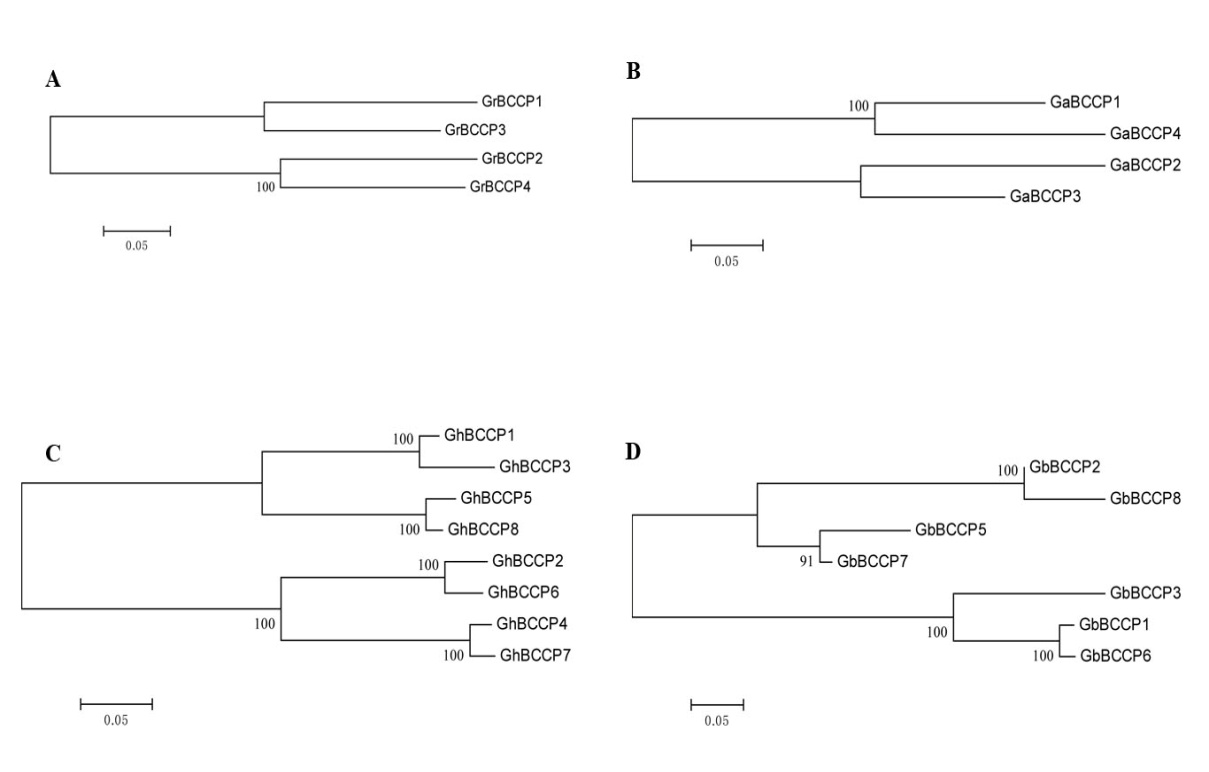
**

**Supplementary Figure 5. Phylogenetic relationships of *BCCP* genes in each of *G*. *raimondii*, *G*. *arboreum, G*. *hirsutum* and *G. barbadense*.** (A) The phylogenetic tree of *Gr*BCCP genes in G. raimondii, (B) The phylogenetic tree of *Ga*BCCP genes in G. arboreum, (C) The phylogenetic tree of *Gh*BCCP genes in *G*. *hirsutum*, (D) The phylogenetic tree and gene structure of *Gb*BCCP genes in G. arboreum.

**Supplementary Table 1. *BCCP* genes used to construct phylegenetic tree in other species.**

| Name | Accession NO. | Species |
| --- | --- | --- |
| *AtBCCP1* | AT2G29750 | *Arabidopsis thaliana* |
| *AtBCCP2* | AT4G36770 | *Arabidopsis thaliana* |
| *BnpBP6* | X90731 | *Brassica napus* |
| *BnpBP7* | X90732 | *Brassica napus* |
| *GmaccB-1* | AF162283 | *Glycine max* |
| *GmaccB-2* | AF271796 | *Glycine max* |

**Supplementary Table 2. Primers for qRT-PCR used in this study.**

| Gene name | Forward primer (5'-3') | Reverse primer (5'-3') |
| --- | --- | --- |
| *GhBCCP1* | GCAGCAGTACATGCCTCAACC | ATGAGAAGAACCAACCGCTTTA |
| *GhBCCP2* | CTTCCTGCTGGTCAAGCATCT | GTACCGGCCATTGGACATTTA |
| *GhBCCP3* | GTGCCGGACTCTGGTAGAAGG | TCATGCCACAATACATGCCTC |
| *GhBCCP4* | CAGCAGGTGCTTGACCAGAAG | TTGCAGCTTAAACAACTTGACTG |
| *GhBCCP5* | TGAGAAGAACCAACCGCTTTA | TGATTCACGAGATATTACGGAGC |
| *GhBCCP6* | CCACTGCCTTGCCTAATTCC | CTGTTGGTGCCCCTGATTTG |
| *GhBCCP7* | ACTTGAGTGGAGGATGAGATGAGC | TTGCAGCCACCGGAACAA |
| *GhBCCP8* | GGCTGACTTGGGAGCTGGA | TGTTATGTCGCAGGCATCCTC |
| *GaBCCP1* | TGCTTCTGCTGCACCTACAA | GGCGGAGGCATTACTGGTAG |
| *GaBCCP2* | TTGCTCTTCCTCCTTGTTCCAA | TGCAACATCCGGTATCTTATTGC |
| *GaBCCP3* | AAACAAACCAGCAAACCCAAAC | CACTGCAAACGACAGCCAGATA |
| *GaBCCP4* | CCACCTTGCCTTTCCTTTCG | AGACATTACTTGATGGCTTAGCAT |
| *GrBCCP1* | TCTTTTGCTCTTCCTCATTGTTC | ATAGTTTTCTTCTCAGTATGCCACT |
| *GrBCCP2* | TCTCCACCGTCTCCTTTCG | TGGGCTTTCACCACTGTTT |
| *GrBCCP3* | ATCTCACAAGGGTTCTTTGTCTC | GACTGTAGTTTGCTTCCCATCA |
| *GrBCCP4* | CTACCTTGCCTTTCCTTTCG | ATGGCTTAGCATCCTGTGGT |
| *GhUBQ7* | GAAGGCATTCCACCTGACCAAC | CTTGACCTTCTTCTTCTTGTGCTTG |

**Supplementary Table 3. Sequence and length details of the identified motifs in cotton BCCP proteins using MEME tool.**

| Motif No. | Motif length (AA) | Sequence |
| --- | --- | --- |
| 1 | 80 | PAKAVGSSHPPLKCPMAGTFYRSPAPGEPPFVKVGDKVQKGQVVCIIEAMKLMNEIEADQSGTVTEILVEDGKSVSVDMW |
| 2 | 74 | DDKRSAEKAIPDAAAISEFMAQVSDLVKLVDSRDITELQLKQSDCELVIRKKEALQQPESASPIVMPQYVPQPT |
| 3 | 29 | LQHGQNSKTVVKAQLNEVAIDGSSNASAA |
| 4 | 57 | SSLSFPCPKISSFLKTNQQTQAHKGSLSLPPTSNFNSKSCLSFGSSIRVPAFSASQW |

**Supplementary Table 4. Nine duplicated *BCCP* gene pairs in *G. raimondii*, *G. arboreum*, *G. hirsutum* and *G. barbadense*.** The alignment length covered >70% of the longer aligned gene, and the amino acid identity between the sequences was >70%, to identify gene duplication events.

| Species | Duplicated gene 1 | Duplicated gene 2 |
| --- | --- | --- |
| *G. raimondii* | *GrBCCP1* | *GrBCCP3* |
|  | *GrBCCP2* | *GrBCCP4* |
| *G. arboreum* | *GaBCCP1* | *GaBCCP4* |
|  | *GaBCCP2* | *GaBCCP3* |
| *G. hirsutum* | *GhBCCP1* | *GhBCCP3* |
|  | *GhBCCP2* | *GhBCCP6* |
|  | *GhBCCP4* | *GhBCCP7* |
|  | *GhBCCP5* | *GhBCCP8* |
| *G. barbadense* | *GbBCCP5* | *GbBCCP7* |

**Supplementary Table 6. The *cis*-elements involved in salt and cold stresses response in the promoter regions of *GrBCCPs*, *GaBCCPs* and *GhBCCPs*.** _indicated no corresponding *cis*-element.

| Gene name | Environmental stress-related element | | | | | | | |
| --- | --- | --- | --- | --- | --- | --- | --- | --- |
|  | HSE | LTR | MBS | TC-rich repeats | ARE | Box-W1 | W box | WUN-motif |
| *GrBCCP1* | - | 1 | 1 | 1 | 1 | - | - | - |
| *GrBCCP2* | 1 | - | - | 1 | 1 | - | - | - |
| *GrBCCP3* | 1 | - | 1 | - | 1 | 1 | 1 | - |
| *GrBCCP4* | 1 | 1 | 1 | 1 | 1 | - | - | - |
| *GaBCCP1* | 1 | - | 1 | 1 | 1 | - | - | - |
| *GaBCCP2* | 1 | - | 1 | - | 1 | - | - | 1 |
| *GaBCCP3* | 1 | 1 | - | 1 | 1 | - | - | - |
| *GaBCCP4* | 1 | - | 1 | 1 | 1 | - | - | - |
| *GhBCCP1* | 1 | - | - | 1 | 1 | - | - | - |
| *GhBCCP2* | 1 | - | 1 | 1 | 1 | - | - | - |
| *GhBCCP3* | 1 | - | 1 | - | - | - | - | - |
| *GhBCCP4* | 1 | - | 1 | 1 | 1 | - | - | - |
| *GhBCCP5* | 1 | - | 1 | - | - | - | - | - |
| *GhBCCP6* | 1 | 1 | 1 | 1 | 1 | 1 | 1 | - |
| *GhBCCP7* | - | 1 | 1 | 1 | 1 | - | - | - |
| *GhBCCP8* | 1 | - | 1 | 1 | 1 | 1 | 1 | - |

HSE: cis-acting element involved in heat stress responsiveness

LTR: cis-acting element involved in low-temperature responsiveness

MBS: MYB binding site involved in drought-inducibility

TC-rich repeats: cis-acting element involved in defense and stress responsiveness

ARE: cis-acting regulatory element essential for the anaerobic induction

Box-W1: fungal elicitor responsive element

W box: wounding and pathogen respons

WUN-motif: wound-responsive element
